# Cancer-oocyte SAS1B protein is expressed at the cell surface of multiple solid tumors and targeted with antibody-drug conjugates

**DOI:** 10.1136/jitc-2023-008430

**Published:** 2024-03-13

**Authors:** Arabinda Mandal, Jagathpala Shetty, Christine A Tran, Walter C Olson, Mriganka Mandal, Bhupal Ban, Eusebio S Pires, Sara J Adair, Todd W Bauer, Craig L Slingluff, John C Herr

**Affiliations:** 1 Department of Surgery, University of Virginia Health System, Charlottesville, Virginia, USA; 2 Department of Biomedical Engineering, University of Virginia School of Medicine, Charlottesville, Virginia, USA; 3 Massachusetts Institute of Technology, Cambridge, Massachusetts, USA; 4 Crossbow Therapeutics Inc, Cambridge, Massachusetts, USA; 5 Department of Cell Biology, University of Virginia School of Medicine, Charlottesville, Virginia, USA; 6 Innovation Ventures, Office for Research, Rutgers University, Piscataway, New Jersey, USA; 7 Department of Obstetrics and Gynecology, University of Virginia Health System, Charlottesville, Virginia, USA

**Keywords:** Cytotoxicity, Immunologic, Epitope Mapping, Immunohistochemistry, Tumor Biomarkers

## Abstract

**Background:**

Sperm acrosomal SLLP1 binding (SAS1B) protein is found in oocytes, which is necessary for sperm-oocyte interaction, and also in uterine and pancreatic cancers. Anti-SAS1B antibody-drug conjugates (ADCs) arrested growth in these cancers. However, SAS1B expression in cancers and normal tissues has not been characterized. We hypothesized that SAS1B is expressed on the surface of other common solid cancer cells, but not on normal tissue cells, and might be selectively targeted therapeutically.

**Methods:**

SAS1B expression in human normal and cancer tissues was determined by immunohistochemistry, and complementary DNA (cDNA) libraries were employed to PCR amplify human SAS1B and its transcripts. Monoclonal antibodies (mAbs) to human SAS1B were generated using mouse hybridomas. SAS1B deletion constructs were developed to map SAS1B’s epitope, enabling the creation of a blocking peptide. Indirect immunofluorescence (IIF) of human transfected normal and cancer cells was performed to assess SAS1B expression. SAS1B intracellular versus surface expression in normal and tumor tissues was evaluated by flow cytometry after staining with anti-SAS1B mAb, with specificity confirmed with the blocking peptide. Human cancer lines were treated with increasing mAb and ADC concentrations. ATP was quantitated as a measure of cell viability.

**Results:**

SAS1B expression was identified in a subset of human cancers and the cytoplasm of pancreatic islet cells. Two new SAS1B splice variants were deduced. Monoclonal antibodies were generated to SAS1B splice variant A. The epitope for mAbs SB2 and SB5 is between SAS1B amino acids 32–39. IIF demonstrated intracellular SAS1B expression in transfected kidney cells and on the cell surface of squamous cell lung carcinoma. Flow cytometry demonstrated intracellular SAS1B expression in all tumors and some normal cells. However, surface expression of SAS1B was identified only on cancer cells. SB2 ADC mediated dose-dependent cytotoxic killing of multiple human cancer lines.

**Conclusion:**

SAS1B is a novel cancer-oocyte antigen with cell surface expression restricted to cancer cells. In vitro, it is an effective target for antibody-mediated cancer cell lysis. These findings support further exploration of SAS1B as a potential therapeutic cancer target in multiple human cancers, either with ADC or as a chimeric antigen receptor-T (CAR-T) cell target.

**WHAT IS ALREADY KNOWN ON THIS TOPIC**

- Sperm acrosomal SLLP1 binding protein (SAS1B) is a zinc metalloproteinase expressed in human oocytes that has also been shown to represent a potential immunotherapeutic target on human uterine cancers and pancreatic cancer cells. However, little is known about SAS1B expression on other solid organ malignancies. Interestingly, we later found that SAS1B is also expressed in normal human pancreatic islet cells, raising the concern for cross-reactivity and toxicity with employing SAS1B as a therapeutic target. Thus, our group sought to investigate our hypothesis that SAS1B is selectively expressed on the surfaces of solid cancer cells, but not on those of normal tissue cells, and that this selective expression may be a promising target for antibody-based therapies for multiple solid tumors.

**WHAT THIS STUDY ADDS**

- To our knowledge, this study is the first to demonstrate SAS1B expression in a variety of solid organ malignancies of various histologies. The most interesting is the unique expression of SAS1B on the cell surface of cancer cells and not normal tissues. This selective expression of surface SAS1B was tested in vitro as a potential immunotherapeutic target in human cancers using an antibody-drug conjugate, in which a strong dose-dependent cytotoxic killing was observed across all tumor cell lines.

**HOW THIS STUDY MIGHT AFFECT RESEARCH, PRACTICE OR POLICY**

- This current paper is novel in that it demonstrates SAS1B to be a candidate immunotherapeutic target in a variety of human solid organ malignancies, many of which do not have very effective therapies. Selectively targeting SAS1B has the potential to have a broad and profound impact on the treatment, and therefore a reduction in mortality, of multiple malignancies. Our findings additionally support further exploration of antibody-based therapies, including antibody-drug conjugates and chimeric antigen receptor-T cell (CAR-T) therapy.

## Background

Antibodies binding to surface antigens on human cancer cells can be effective therapeutics by modulating function or lysing these cells. A challenge for antibody therapies for solid tumors is the paucity of tumor-specific cell surface antigens. Of 13 Food and Drug Administration-approved antibody-drug conjugates (ADCs), only four are approved to treat solid tumors.^1^ Even these targets are expressed on some normal tissues. The paucity of specific surface antigens on solid tumors limits the success of chimeric antigen receptor T (CAR-T) cell therapy for solid tumors.^2^ Our group has reported that SAS1B (sperm acrosomal SLLP1 binding protein, ovastacin, astacin-like, ASTL) is a zinc metalloproteinase that binds to sperm acrosomal SLLP1 binding protein, expressed in human oocytes.^3 4^ We also demonstrated that SAS1B represents a potential immunotherapeutic target for human uterine and pancreatic cancers. Antibodies targeting SAS1B arrested their growth in vitro.^5 6^ Because SAS1B is expressed by mature oocytes and human cancer cells, it represents a novel class of cancer-oocyte proteins.

The apparent restriction of SAS1B to growing oocytes among normal tissues suggests that tumor cells expressing surface SAS1B might be selectively targeted. Data on SAS1B expression on other solid malignancies and normal tissues are limited. We now have monoclonal antibodies (mAbs) useful for immunohistochemistry (IHC) and flow cytometry, enabling more detailed analyses of intracellular and surface SAS1B expression in human normal and cancer cells.

In this manuscript, we report the development and characterization of anti-SAS1B mAbs, including the identification of a blocking peptide that enables the validation of antibody specificity. We hypothesized that SAS1B is tumor-specific and is not expressed on normal tissue cells. We screened cancer and normal cells by IHC and evaluated them with anti-SAS1B mAbs, along with a blocking peptide. We found that SAS1B is expressed intracellularly in multiple human cancers and normal tissues. However, we found that the surface expression of SAS1B is tumor-specific. We provide evidence that an ADC targeting surface SAS1B can lyse solid tumor cell lines. These data provide evidence that SAS1B is a promising target for antibody-based therapies for solid tumors.

## Materials and methods

### Tissue microarrays/IHC of human normal and cancer tissues

Tissue microarrays (TMAs) of normal and cancer tissues (common cancer 1B and 2B) from the University of Virginia (UVA) Biorepository and Tissue Research Facility were stained with anti-SAS1B rabbit polyclonal antibody (pAb), which was generated by this group.^3^ Standard TMA technologies were used and performed by the Cooperative Human Tissue Network.^7^

### Human SAS1B splice variants

SAS1B splice variants (SV) identified from PCR products were amplified from human ovary complementary DNA (cDNA) and also from RNA isolated from: (1) uterine cancer cell line SNU539 (malignant mixed Mullerian tumor (MMMT)) (Seoul National University, Seoul, South Korea); (2) pancreatic ductal adenocarcinoma mouse xenograft tumor samples; (3) human primary head and neck squamous cell carcinoma samples. Pancreatic tumor xenografts originated as surgical human cancer specimens that were implanted into nude mice pancreases and propagated in vitro; tumor RNA interrogated here was from ~F5 generation. PCR products were subcloned and sequenced. Structural features were deduced using I-TASSER. Identification was confirmed by subcloning and sequencing PCR amplicon.

### Anti-human SAS1B mAbs generation

To have an antibody to determine cell surface and/or intracellular SAS1B expression in human cancer and normal cells, His-tagged purified recombinant human SAS1B (rhSAS1B) splice variant A (SV-A) was prepared as an immunogen and used to produce mAbs.^3 8^ This was performed by the UVA Antibody Engineering and Technology Core, which was responsible for mAb batch production. BALB/c mice were immunized subcutaneously at 3-week intervals with 30 µg of purified rhSAS1B in complete Freund’s adjuvant for initial injection and incomplete Freund’s adjuvant for subsequent injections. A final intrasplenic booster was administered three days before fusion, using recombinant protein without adjuvant. Splenic cells from immunized mice were fused with mouse myeloma cell line Sp2/0-Ag14 at a 5:1 ratio, using 50% polyethylene glycol (PEG 4,000) as the fusogen, followed by suspension and selection in hypoxanthine and thymidine containing Iscove’s Modified Dulbecco’s Medium. Antibody-producing parent hybridomas were cloned at least twice by limiting dilutions. Supernatants from sequentially cloned populations were screened against rhSAS1B in 50 mM carbonate-bicarbonate buffer (pH 9.5) by solid phase ELISA. Positive clones were expanded in 24-well plates and 75 cm^2^ flasks (Nalge Nunc, Rochester, New York, USA), and grown for antibody purification in 500 mL gas permeabilized bags.^9^ The mAbs were assessed for reactivities and specificities against recombinant His-tagged proteins via western blotting. Those with the best reactivities and specificities were used in later experiments.

### Immunoprecipitation and tandem mass spectrometry analysis

HEK293T cells (UVA Core Tissue Culture Facility, Charlottesville, Virginia, USA) were seeded in T75 flasks in complete DMEM/F-12 media (Thermo Fisher Scientific, Waltham, Massachusetts, USA) to ~85% confluency before transfection with rhSAS1B SV-A in pcDNA3.1/V5-His-Topo plasmid. Flasks were transfected with 20 µg DNA in 2.25 mL of Opti-MEM media (Life Technologies, Carlsbad, California, USA) containing 40 µL of TurboFect (Thermo Fisher Scientific) for 72 hours. Cells were washed and scraped in Hank’s balanced salt solution (Life Technologies) at 4°C. Pellets were extracted in radioimmunoprecipitation assay (RIPA) buffer (Thermo Fisher Scientific) containing complete protease inhibitor (Roche, Mannheim, Germany) on ice for 1 hour. The supernatant was collected after centrifugation (16,000×g) and used for immunoprecipitation using magnetic beads complexed with Protein G (Life Technologies).

Anti-SAS1B mAbs (SB2 and SB5), a non-specific mAb (3A4, anti-Ca-binding tyrosine phosphorylation regulated protein (CABYR)),^10^ or no mAb in phosphate buffer saline (PBS) with 0.05% Tween 20 (PBST, Thermo Fisher Scientific) were incubated with magnetic Dynabeads Protein G (Thermo Fisher Scientific) at room temperature (RT) following manufacturing’s protocol, then washed in PBST. Dynabeads-mAb complexes were exposed to rhSAS1B-transfected HEK293T extract in RIPA buffer at 4°C for 30 min and washed in wash buffer (×4) with protease inhibitor. Beads were eluted in Laemmli sample buffer, heated at 98°C for 10 min, and analyzed by SDS-PAGE, Coomassie staining, western analyses using anti-His tag mAb, and mass spectrometry (MS)/MS analysis.

### SAS1B transfection in HEK293T cells

HEK293T cells were cultured in DMEM/F-12, 4-(2-hydroxyethyl)-1-piperazineethanesulfonic acid (HEPES) (Life Technologies) containing 10% fetal bovine serum (FBS, heat-inactivated at 56°C for 30 min), MEM non-essential amino acids (0.1 mM, Thermo Fisher Scientific), and sodium pyruvate (1 mM) at 37°C in a humidified incubator with 5% CO_2_. For transient transfection, HEK293T cells were seeded at 0.5×10^6^ cells per T75 vented culture flask (Genesee Scientific, San Diego, California, USA) 1-day prior to transfection. Plated cells were transfected with 5 or 20 µg of SAS1B-pcDNA3.1/V5-His topo plasmid DNA using TurboFect Transfection Reagent (Thermo Fisher Scientific) following manufacturing’s instructions. Cells were harvested 48 or 72 hours post-transfection, washed with PBS, used for fluorescence microscopy or preparation of protein lysates, or frozen at −80°C until further use.

### Mapping SAS1B epitopes by western blotting of recombinant proteins from deletion constructs

Monoclonal antibody specificities were tested with rhSAS1B proteins made in *Escherichia coli*, HEK293T, and SNU539 protein extracts. Methods for bacterial and mammalian expression of SAS1B protein fragments are aforementioned. Bacterially expressed rhSAS1B protein (125 ng/lane) was solubilized in 2× Laemmli sample buffer (Bio-Rad Laboratories, Hercules, California, USA), while untransfected/transiently transfected HEK293T cell extracts were made in Celis buffer.^11^ The supernatant was reconstituted in 6× Laemmli buffer (~60 µL/lane). SNU539 extracts were made in RIPA buffer followed by 6× Laemmli buffer (~60 µL/lane). Proteins were resolved on 12.5% SDS-PAGE Criterion gels (Bio-Rad Laboratories), blotted to nitrocellulose membrane (0.2 µm, Bio-Rad Laboratories), and blocked with 5% non-fat dry milk in PBST for 45 min at RT. Blots were probed with primary antibody generated from the hybridomas (1 µg/mL for rhSAS1B, 2 hours incubation; 3 µg/mL for SNU539, overnight incubation), washed for 10 min in PBST 3×, and incubated with peroxidase-conjugated goat anti-mouse IgG secondary antibody (1:5,000) for 1 hour. Blots were washed in PBST (10 min, 2×) and PBS (10 min, 3×), and developed in tetramethylbenzidine (TMB) peroxidase substrate (Kirkegaard and Perry Lab, Gaithersburg, Maryland, USA).

SAS1B SV and deletion constructs were expressed in HEK293T cells for mAb western blot screening. Eight SAS1B expression constructs were generated to encompass various SAS1B domains (table 1). DNA fragments for each SAS1B insert were generated by PCR from human SAS1B SV-A and splice variant C (SV-C) plasmids. SAS1B DNA fragments for constructs 1, 2, and 4–8 were fused in-frame with pcDNA 3.1/V5-His-topo vector (Invitrogen, Waltham, Massachusetts, USA) by topocloning. The vector carried a C-terminal V5 epitope followed by a polyhistidine (6× His) tag. SAS1B DNA fragment for construct 3 was subcloned into pSec Tag/FRT/V5-His-topo vector (Invitrogen), which carried an N-terminal murine Ig κ-chain leader sequence, in addition to the pcDNA 3.1/V5-His-topo vector. PCR and DNA sequencing confirmed constructs. Table 1 includes primers for PCR amplification of the inserts.

**Table 1:** Primers used for PCR amplification of SAS1B and development of deletion constructs

Number	Construct	Primers
1	SAS1B-SV-A (1–431) – pcDNA 3.1/V5-His-topo	Forward: GAT ATG GAG GGT GTA GGG GGT CTC TGG
		Reverse: ATC TTC GGA CAT CCC CTT GAA ATG ATT
2	SAS1B-SV-C (1–436) – pcDNA 3.1/V5-His-topo	Forward: GAT ATG TCC TGC TGT CTG GTC TCA CCG
		Reverse: ATC TTC GGA CAT CCC CTT GAA ATG ATT
3	SAS1B-SV-A (164–431) – pSec Tag/FRT/V5-His-topo	Forward: CAG GTG GTC TCC CTG GCG CCC ACG TGT
		Reverse: ATC TTC GGA CAT CCC CTT GAA ATG ATT
4	SAS1B-SV-A (24–431) – pcDNA 3.1/V5-His-topo	Forward: GAT ATG GCG CCC CTG GCC TCC AGC TGC
		Reverse: ATC TTC GGA CAT CCC CTT GAA ATG ATT
5	SAS1B-SV-A (32–431) – pcDNA 3.1/V5-His-topo	Forward: GAT ATG GGA GCC TGT GGT ACC AGC TTC
		Reverse: ATC TTC GGA CAT CCC CTT GAA ATG ATT
6	SAS1B-SV-A (40–431) – pcDNA 3.1/V5-His-topo	Forward: GAT ATG GAT GGC CTC ACC CCT GAG GGA
		Reverse: ATC TTC GGA CAT CCC CTT GAA ATG ATT
7	SAS1B-SV-A (48–431) – pcDNA 3.1/V5-His-topo	Forward: GAT ATG CAG GCC TCC GGG GAC AAG GA
		Reverse: ATC TTC GGA CAT CCC CTT GAA ATG ATT
8	SAS1B-SV-A (55–368) – pcDNA 3.1/V5-His-topo	Forward: GAT ATG TTC CTG CAA TTA ACC AAA GGG
		Reverse: GGA AGC TAG GGT CTG AGG CTG CCT

SAS1B, sperm acrosomal SLLP1 binding protein; SV-A, splice variant A; SV-C, splice variant C.

### Indirect immunofluorescence of human normal and cancer cell lines for SAS1B

Plasmid made from full-length SAS1B, cloned into pcDNA3.1/V5/His-topo vector (SV-A), was used for transfection in HEK293T cells at ~50–70% confluence. Cells were seeded on 24-well plates containing poly-d-lysine coated 12 mm round coverslips and transfected with TurboFect Transfection Reagent for 48 hours at 37°C (1 µg DNA/100,000 cells/well). Cells were fixed with 4% paraformaldehyde (Alfa Aesar, Ward Hill, Massachusetts, USA) in PBS for 15 min, washed in PBS 3×, permeabilized in 100% methanol for 15 min, washed in PBS 3×, and blocked in culture media containing 5% normal goat serum for 30 min. Cells were incubated with SAS1B mAbs (10 µg/mL) or rabbit anti-V5 epitope tag antibody-DyLight 488 conjugated (5 µg/mL, Rockland Immunochemicals, Philadelphia, Pennsylvania, USA) for 1 hour. SAS1B-mAbs complexes were localized with Alexa Fluor 594 conjugated F(ab)_2_ goat anti-mouse IgG antibody (5 µg/mL, Jackson ImmunoResearch, West Grove, Pennsylvania, USA) for 1 hour in a blocking buffer. Cover slips were mounted with SlowFade Diamond containing DAPI (Molecular Probes, Eugene, Oregon), sealed with nail polish, and visualized in Zeiss LSM 700 confocal and axiovert 200 fluorescence microscopes (Carl Zeiss, GmbH, Oberkochen, Germany).

The SAS1B^+^ SNU539^5^ was grown to ~85% confluency in RPMI-1640 media (Life Technologies) containing 10% FBS, 10 mM HEPES, and 2 mM L-Glutamine (Life Technologies) in a T75 flask at 37°C with 5% CO_2_. Cells were detached using 4 mL of Accutase (BioLegend, San Diego, California, USA) at 37°C for 7–8 min, followed by the addition of 10 mL of media, and spun for 2 min at 70×g. The pellet was suspended in 10 mL of media, counted using a disposable Hemocytometer (InCyto C-Chip DHC-N01-5, Fischer Scientific, Hampton, New Hampshire, USA), and seeded on collagen-coated coverslips (Neuvitro, Vancouver, Washington, USA) in 24-well plates at 20,000 cells in 1 mL media, which was replaced after 24 hours. After seeding for 48 hours, cells were kept on ice for ~8 min, incubated with mAbs (20 µg/mL) in media with 15 mM NaN_3_ for 1 hour on ice, washed in 1 mL of cold media with 15 mM NaN_3_×3, and incubated with Alexa Fluor 488 conjugated F(ab)_2_ goat anti-mouse IgG antibody (5 µg/mL) in cold media with 15 mM NaN_3_ for 1 hour on ice. Cells were washed in cold 1 mL media with 15 mM NaN_3_×3, fixed in 4% paraformaldehyde in PBS for 20 min on ice, washed in 1 mL H_2_O, mounted in SlowFade Diamond Antifade with DAPI (Life Technologies), and imaged using Zeiss LSM 700 confocal microscopy.

### Intracellular and surface staining of human cancer cell lines and normal tissues for flow cytometry

Specimens were obtained from commercial sources or as surgical specimens at UVA under Institutional Review Board for Health Sciences Research (IRB-HSR) #10598/13529 (online supplemental table 1). Skin fibroblast and renal cell primary cell cultures were established in RPMI-1640 supplemented with L-glutamine, non-essential amino acids, sodium pyruvate, penicillin–streptomycin (Gibco-Life Technologies, Thermo Fisher Scientific), and 10% FBS. Primary cultures of cardiac myocytes, aortic endothelium, and skeletal muscle cells (PromoCell, Heidelberg, Germany) were cultured according to the supplier’s instructions. Tumor cell lines were grown in T75 flasks to 80–90% confluency and detached from flasks with Accutase (Millipore-Sigma, Burlington, Massachusetts, USA) after incubating 5–10 min at 37°C. On receipt from the supplier (Prodo Laboratories, Aliso Viejo, California, USA), pancreatic islets were suspended in Corning Miami Media (MediaTech, Manassas, Virginia, USA) containing 10% FBS, L-glutathione (Sigma-Aldrich, St. Louis, Missouri, USA), antibiotics, HEPES, and L-glutamine (Life Technologies), transferred to wells (6-well cluster dish, CoStar, Washington, DC, USA), and incubated at 37°C overnight. Before staining, islets were collected, washed, and dissociated in Accutase by pipetting every 5 min during a 15 min incubation period at 37°C. Vials of lymphoid cells from liquid nitrogen were thawed in a 37°C water bath and transferred to RPMI-1640 thaw media containing DNAse (100 Kunitz units/mL, Worthington Biochemical, Lakewood, New Jersey, USA) and 10% FBS. Cells were washed twice in RPMI-1640+10% FBS (RPMI/FBS) and rested for 1–2 hours in RPMI/FBS. Cells rested in RPMI/FBS for 1.5–2 hours in 5% CO_2_ while cell counts were performed in Trypan blue. After adjusting cell concentrations to 1×10^6^ cells/mL, 200 µL of cells were distributed to each well of a Corning 96-well round bottom polypropylene microplate (Product #3879, Corning, New York, USA) and placed on ice.

10.1136/jitc-2023-008430.supp1 Supplementary data

Peptide blocking was performed.^6^ Peptides (Atlantic Peptides, Lewisburg, Pennsylvania, USA) from SAS1B’s N- (blocking: aa24-42, APLASSCAGACGTSFPDGL) and C- (control: aa377–391, GAPGVAQEQSWLAGV) termini were added in fivefold excess to SB2 and SB5 purified mAbs. After incubating for 1–1.5 hours at 4°C, 100 µL of peptide-treated mAbs were added to 100 µL of cells and incubated for 45 min at 4°C.

To prepare cells for flow cytometry, cells were washed free of serum-containing media in PBS, then labeled with Live/Dead Fixable Dye Aqua (Molecular Probes, Thermo Scientific) according to manufacturing’s directions. Aqua was removed by washing twice in PBS containing 10% heat-inactivated FBS and 0.1% sodium azide. Cells were distributed in individual wells of a 96-round bottom well assay plate (CoStar). To assess for intracellular expression of SAS1B in cells, cells were fixed in formaldehyde and permeabilized in saponin using Cytofix/Cytoperm buffers (BD Biosciences, Franklin Lakes, New Jersey, USA). Cell permeabilization allowed for anti-SAS1B mAbs to enter cells and bind to intracellular SAS1B. Cells used to assess surface SAS1B expression were left intact and viable. Thus, anti-SAS1B mAbs only stained surface-expressed SAS1B on viable cells, but stained intracellular and surface SAS1B on permeabilized cells. For intracellular and surface staining, the following primary antibodies at their final concentrations in FACS buffer (RPMI/FBS+0.1% NaN_3_) were added to designated wells: anti-SAS1B (SB2 or SB5; 10 µg/mL), anti-HLA-ABC (w632; 1 µg/mL), isotype controls mouse IgG2b (10 µg/mL; Thermo Fisher Scientific Cat# 015-000-003) or IgG (Jackson ImmunoResearch), or anti-CABYR (10 µg/mL). Cells were incubated for 45 min at 4°C and washed thrice with Cytoperm or FACS buffer for intracellular or surface staining, respectively. Secondary antibody fluorescein isothiocyanate-sheep anti-mouse IgG-F(ab’)2 (1 µg/mL; MP Biomedicals, Solon, Ohio, USA) in Cytoperm or FACS buffer for intracellular or surface staining, respectively, was added to each well. Cells were washed thrice with either Cytoperm or FACS buffer for intracellular or surface staining, respectively. Cells for intracellular staining were washed again twice with FACS buffer. Cells for surface staining were fixed with paraformaldehyde. Cells were acquired on a Canto II Flow Cytometer (BD Biosciences). Data were analyzed using FlowJo V.10.8.1 (BD Biosciences).

### ADC assay on cell viability/cytotoxicity

Four cancer cell lines were tested: M1 (renal cell carcinoma), A549, NCI-H226 (ATCC, Manassas, Virginia, USA), and SNU539. Human cancer cells (~750–1500) in 90 µL of complete media were seeded per well in white (opaque-walled) 96-well polystyrene plates with clear bottoms (Corning) 20–24 hours before ADC treatment. The ADC used in this study was the secondary ADC Fab-αMFc-CL-DMDM (Moradec, San Diego, California, USA). A Fab fragment of anti-mouse IgG Fc-specific antibody is conjugated to Duocarmycin DM (DMDM) by a cleavable linker. The linker can be cleaved by cathepsins intracellularly.^12^ This ADC was diluted to 150 nM in complete media. The following primary antibodies were used: SB2, SB5, 3A4 (negative control), and AY13 (anti-epidermal growth factor receptor (EGFR)) (positive control). These antibodies were serially diluted (1000, 100, 10, 1, 0.1, 0.01, 0.001, and 0.0 nM) with media containing the Fab-αMFc-CL-DMDM (150 nM) and incubated at RT for 1–1.5 hours. The primary and secondary antibody complexes in 10 µL of volume were added to 90 µL of media in each well in triplicate and incubated at 37°C with 5% CO_2_ for 4 days. The percent of viable cells in culture was determined by quantitating ATP present, indicating the presence of metabolically active cells, using CellTiter-Glo V.2.0 Luminescent Cell Viability assay system (Promega, Madison, Wisconsin, USA). Media was removed from cells, which were incubated with 200 µL of 1× CellTiter-Glo V.2.0 in Dulbecco’s PBS for 20–30 min in darkness. ATP levels in the plates were read as relative luminescence unit (RLU) using BioTek Cytation3 multi-mode reader (BioTek, Winooski, Vermont, USA). The viability of control live cells (100%) was determined by averaging the nine wells in terms of total cellular ATP (RLU) containing the constant 15 nM Fab-αMFc-CL-DMDM and 0.0 nM of primary mAb for each antibody. The effect of anti-SAS1B mAbs without DMDM conjugated to goat anti-mouse Fab fragment (isotype IgG2b, Jackson ImmunoResearch) on cell viability was also determined.

## Results

### SAS1B protein expression in human cancer and normal tissues

**Figure 1:**
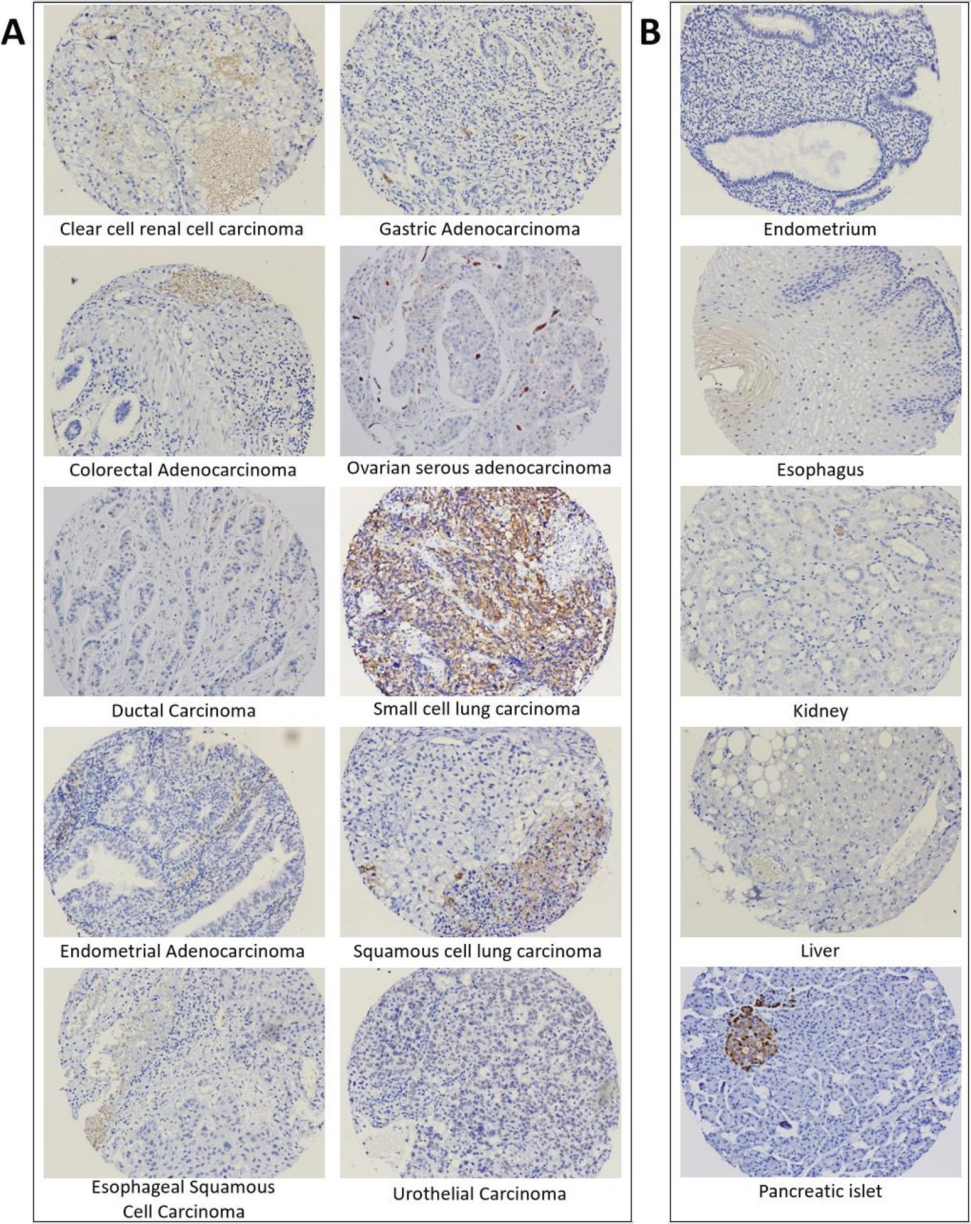
Representative immunohistochemistry sections of (A) human cancer and (B) normal tissues stained with anti-SAS1B rabbit polyclonal antibody. Brown or gray nuclear staining is positive for SAS1B expression. SAS1B, sperm acrosomal SLLP1 binding protein.

SAS1B expression was assessed by IHC with a rabbit anti-SAS1B pAb, which detected SAS1B expression in a subset of human cancers of diverse histologies (figure 1A). Staining was cytoplasmic or nuclear. A definitive assessment of surface staining is not evaluable by IHC. Normal tissue samples were included in TMAs. SAS1B expression was not demonstrated in normal tissues included in TMAs, with the exception of pancreatic islet cells (figure 1B). All core samples had SAS1B^+^ pancreatic islets (data not shown). These data largely confirmed low expression in normal tissues, but expression by pancreatic islets raised concern for potential toxicity if SAS1B were targeted in cancer therapy. To better understand SAS1B expression in normal and cancer cells, intracellular and surface staining was pursued using an mAb to a specific common SAS1B SV, with confirmation of specificity with a blocking peptide.

### Human SAS1B splice (transcript) variants and structures

**Figure 2:**
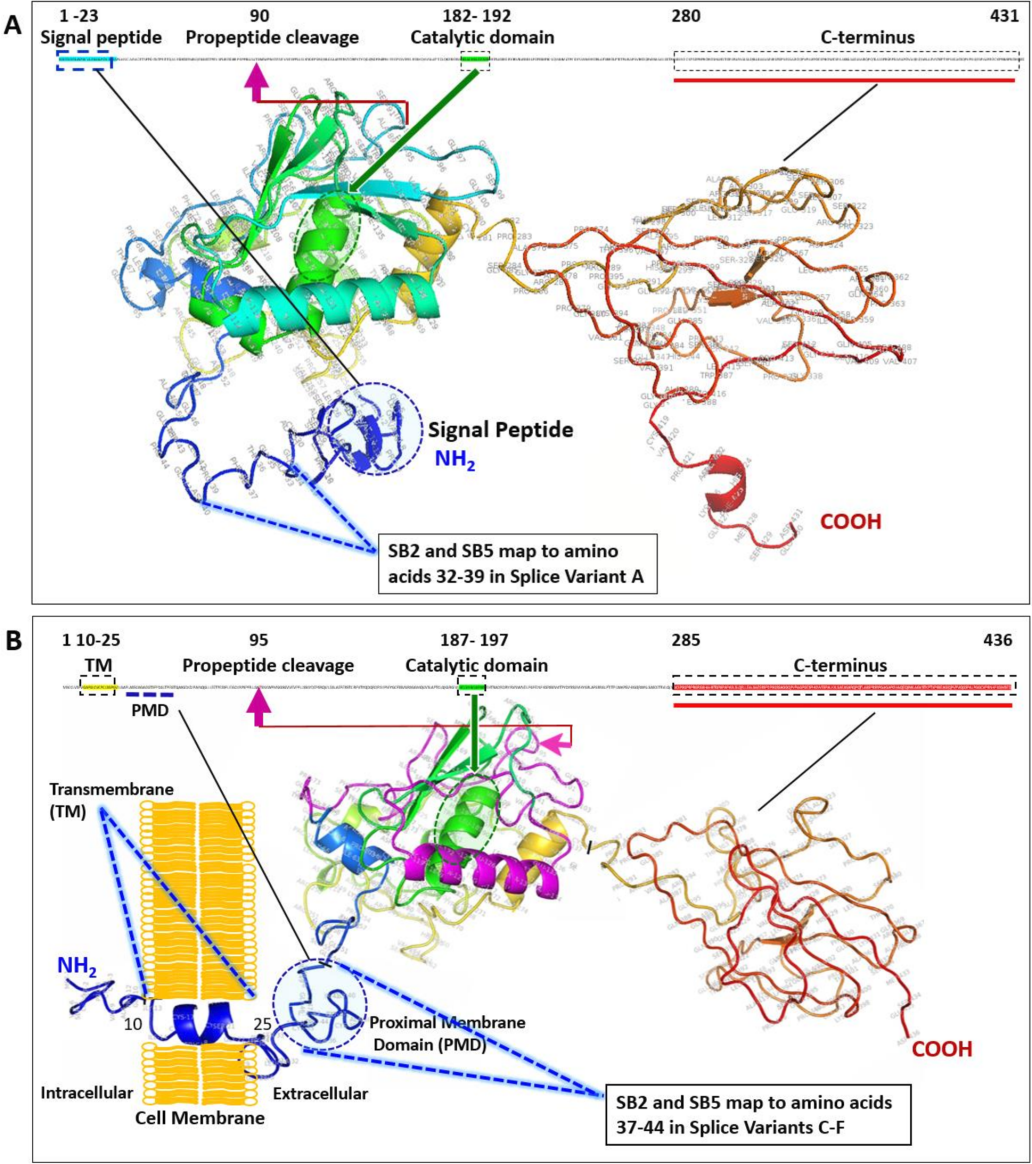
Structure of (A) splice variant A and (B) splice variant C, predicted using I-TASSER.

SAS1B splice variants (SV) were identified in the human ovary. In addition to the known human SAS1B SV, SV-A (NM_001002036), two novel transcript variants were identified: splice variant B (SV-B) (OR231285) and SV-C (OR019679). SV-B was identified by sequencing data generated by subcloning PCR products from the SV-A primer set. SV-A and SV-C were identified from PCR of ovarian cDNA and RNA of multiple human cancers (described in Materials and methods). SV-B and SV-C matched the predicted automated computationally analyzed variants in GenBank (XP_011509509 and XP_011509507, respectively). SV-C sequencing revealed a different coding exon 1 sequence from that of SV-A. SV-B demonstrated an 18 amino acid deletion in exon 7 at the N-terminus. Structural features were deduced for SV-A (figure 2A) and SV-C (figure 2B). SV-A and SV-C are predicted to be intracellular and transmembrane, respectively. Much remains unknown about the structure and localization of each SV and their expression levels in normal and cancer cells. DNA sequences were identified from the aforementioned RNA sources (Materials and methods), confirming the presence of SV-B in different tumor types.

### SB2 and SB5 mAbs specificity to SAS1B

**Figure 3:**
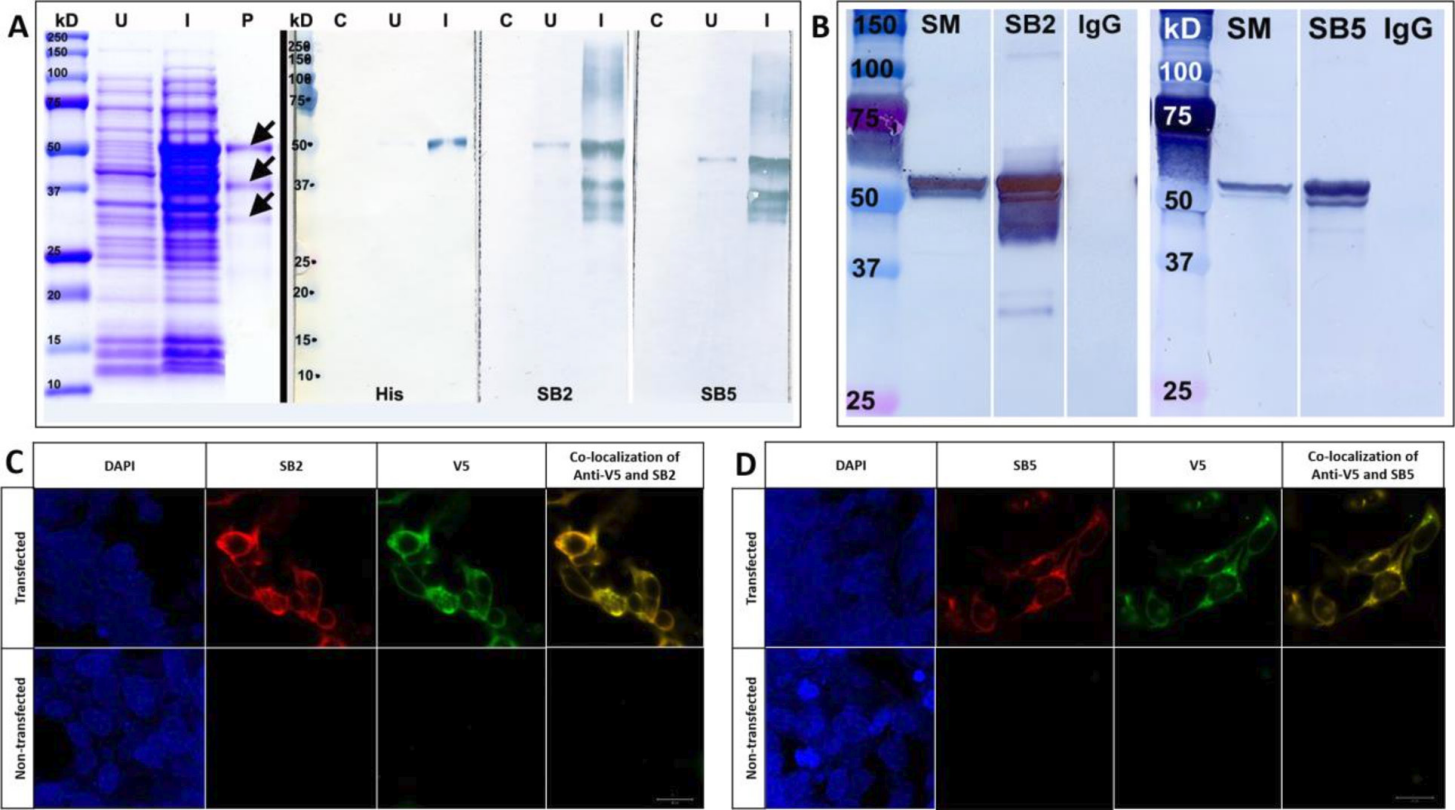
(A) Coomassie staining (left panel) and western blots (right panels) of SAS1B protein expression in *Escherichia coli* probed with anti-His tag or anti-SAS1B mAbs SB2 or SB5. C—control, *E. coli* not transformed with the SAS1B plasmid; U*—E. coli* containing SAS1B plasmids uninduced with IPTG; I—SAS1B transformed *E. coli* induced with IPTG; P—purified SAS1B proteins (black arrows). Both SB2 and SB5 along with anti-His tag mAb detected the ~50 kD SAS1B protein and the breakdown products in the induced *E. coli* extract. (B) Immunoprecipitation of SAS1B from HEK293T cells transfected with SV-A expressed plasmid using SAS1B mAbs SB2 and SB5 immunoprecipitation Western blots. IgG—immunoprecipitation using IgG isotype control; SM—immunoprecipitation starting material of transfected HEK293T cells with SAS1B SV-A plasmid. The western blots show prominent ~50 kD bands pulled down by both SB2 and SB5 mAbs detected by anti-His tag mAb. With regard to SB2, there are also a smear and lower molecular weight bands. All of these bands correlate with the SAS1B protein seen in lanes I and P in the Coomassie stain. (C–D) Indirect immunofluorescence analyses of SAS1B SV-A transfected (top panels) and non-transfected (bottom panels) HEK293T cells. Cells were stained with nuclear stain DAPI, anti-V5 tag antibody conjugated to fluorescein isothiocyanate, and either (C) SB2 or (D) SB5 mAbs along with goat anti-mouse IgG conjugated to Alexa Fluor 594. Stained cells were visualized under a Zeiss LSM 700 confocal microscope, confirming transfection and cytoplasmic co-localization of SAS1B with SB2 and SB5 epitopes and C-terminal V5 epitopes. Legend scale represents 20 µm. DAPI, 4′,6-diamidino-2-phenylindole; IPTG, isopropyl ß-D-1-thiogalactopyranoside; mAbs, monoclonal antibodies; SAS1B, sperm acrosomal SLLP1 binding protein; SV-A, splice variant A.

Murine mAbs to SAS1B were induced by immunization with SAS1B SV-A. Seven mAbs (SB1-SB7) were screened from hybridomas. To assess their specificities, rhSAS1B was expressed in *E. coli* and evaluated by western blot before and after induction with isopropyl ß-D-1-thiogalactopyranoside (IPTG)-inducible lacUV5 promoter. Coomassie staining on purified *E. coli*-expressed rhSAS1B demonstrated a ~50 kD band and two 25–37 kD bands in purified rhSAS1B samples (figure 3A). These bands likely represent SAS1B protein and fragments, as they were also detected by SB2 and SB5 mAbs in induced *E. coli* extract. SAS1B was expressed in HEK293T cells, immunoprecipitated with SB2 and SB5 mAbs, and detected with anti-His tag mAb (figure 3B), consistent with the findings with Coomassie staining and bacterial western blotting (figure 3A). A Coomassie stained ~50 kD SB5 immunoprecipitated band was microsequenced, recovering 12 peptides (33% of SAS1B protein, data not shown). These findings validate the initial specificity of SB2 and SB5 mAbs to SAS1B.

### Cytoplasmic SAS1B protein localization in SAS1B-transfected HEK293T cells

SAS1B-transfected HEK293T cells were stained with an anti-V5 tag antibody to SAS1B’s C-terminus and with SB2 or SB5 mAb to determine cytoplasmic expression. Staining demonstrated prominent immunofluorescence and co-localization of the anti-V5 tag and SB2 or SB5 signal, indicating transfection and cytoplasmic localization of SAS1B protein (figure 3C,D). Non-transfected HEK293T cells were stained as negative controls. Cytoplasmic staining was not detected (figure 3C,D).

### Cell surface SAS1B protein localization in live human tumor cell lines

**Figure 4:**
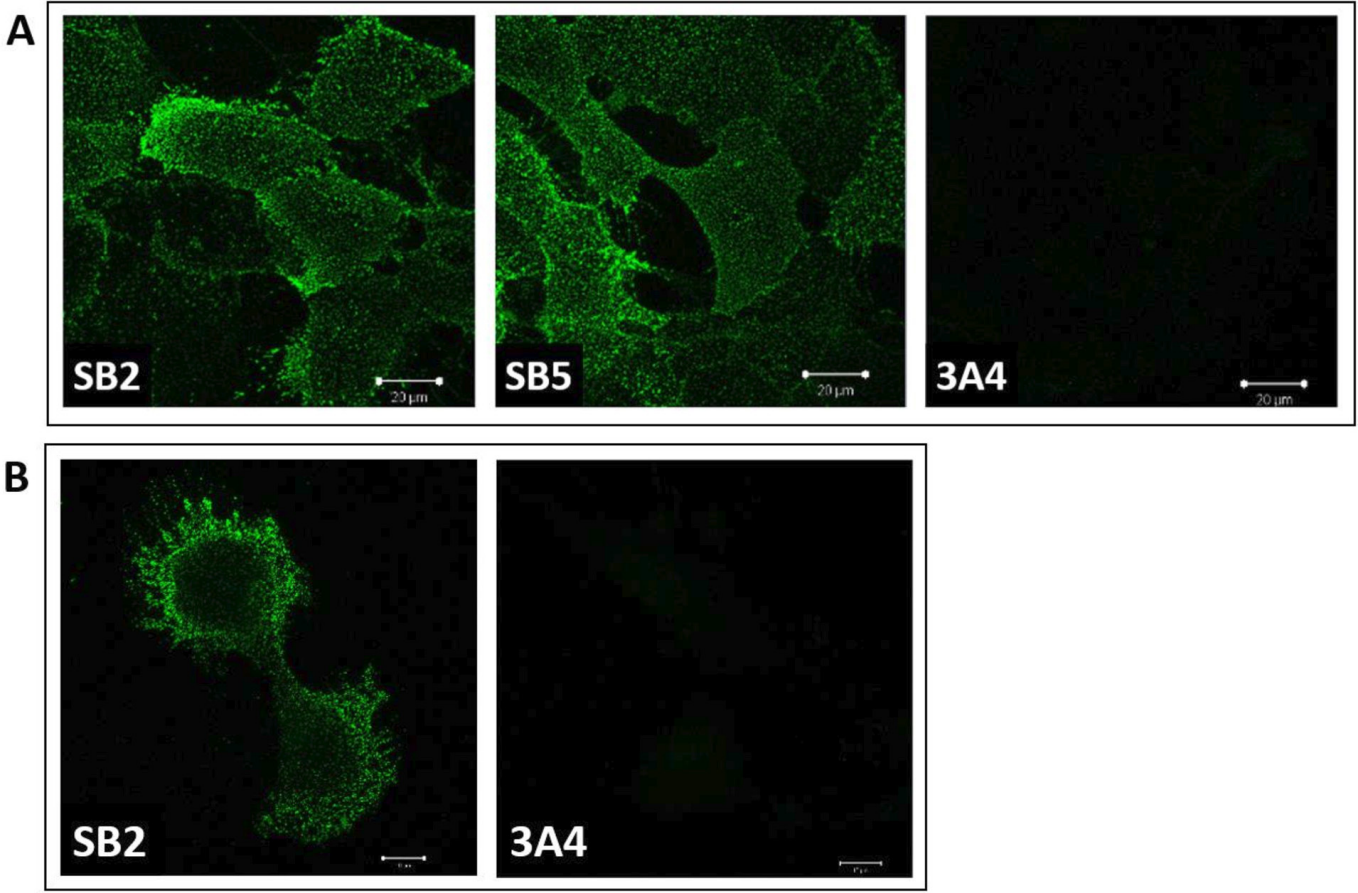
(A) Viable human SNU539 cells were stained with SB2 and SB5 mAbs, in addition to 3A4, an mAb to the intracellular cancer-associated protein, Ca-binding tyrosine phosphorylation regulated protein (CABYR). (B) Viable H226 cells were stained with SB2 and 3A4 mAbs. Sperm acrosomal SLLP1 binding protein cell surface expression is demonstrated by punctate staining. Legend scale represents 20 µm. mAbs, monoclonal antibodies.

This group has previously shown surface SAS1B expression in SNU539.^5^ To test whether SB2 or SB5 stains surface SAS1B on SNU539, viable cells were stained with SB2 or SB5, or with 3A4 (which recognizes the intracellular protein, CABYR (negative control)).^5 10^ Cells were visualized under indirect immunofluorescence (IIF), which demonstrated prominent punctate surface staining with SB2 and SB5 mAbs. 3A4 surface staining was not demonstrated (figure 4A). Live human H226 cells were stained and visualized under IIF. SB2 surface staining was identified (figure 4B).

### Epitope mapping of SAS1B protein

**Figure 5:**
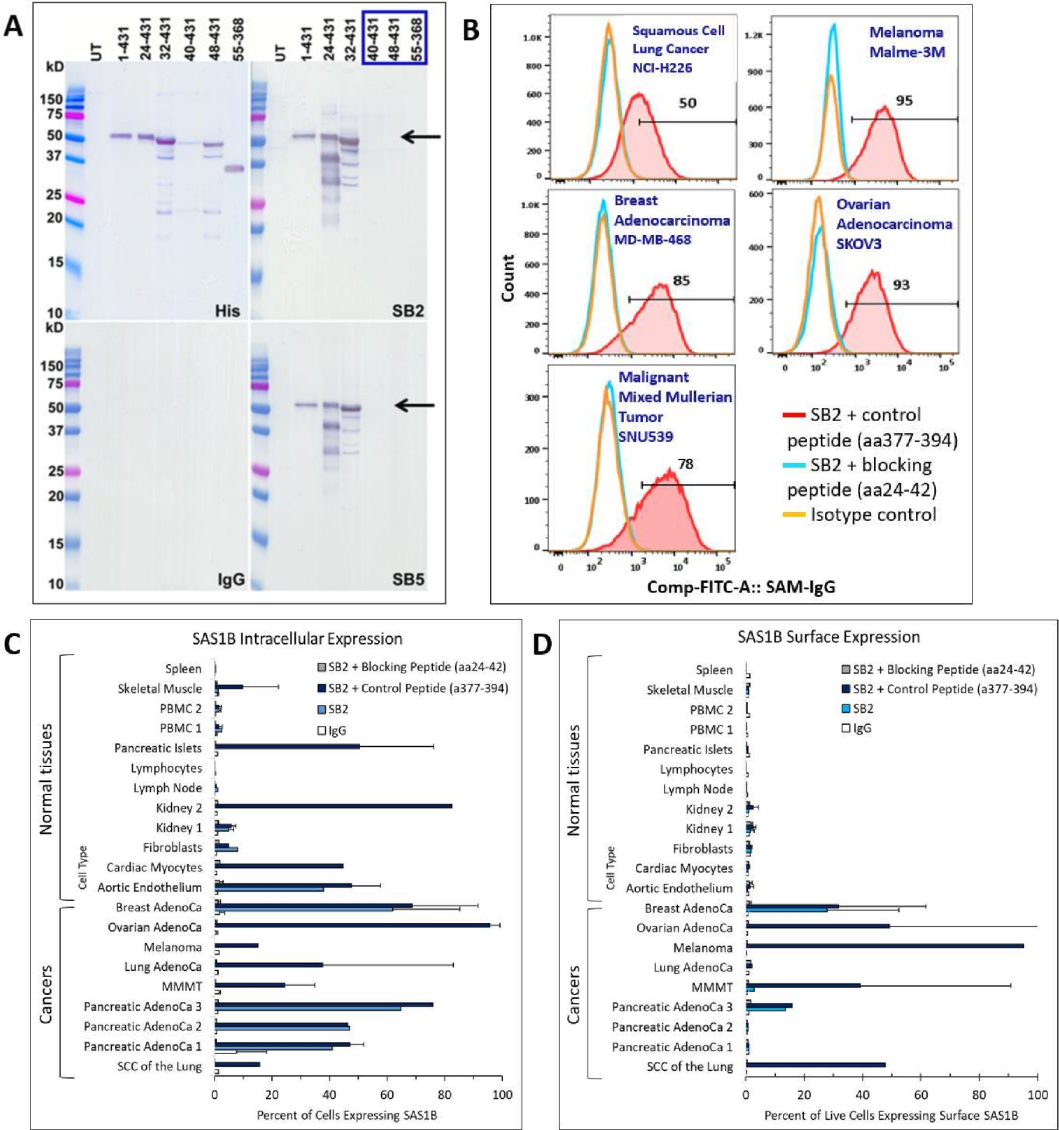
(A) Epitope mapping of SAS1B protein. SB2 and SB5 mAbs demonstrated western immunoreactivity to recombinant human SAS1B with bands weighing approximately ~50 kD (black arrows). SAS1B protein is not detected between amino acids 40–431 (box). SAS1B protein was detected with the anti-C-terminus histidine tag (positive control). SAS1B protein was not detected with IgG (isotype control). (B) Evaluation of SAS1B surface expression in multiple human cancer cell lines by flow cytometry. Individual panels represent a singleton assay for an individual cancer cell line. Cells were stained with SB2 mAb and a negative control peptide (aa377–394) (red curves) or a blocking peptide (aa24-42) (blue curves), and an isotype control (orange curves). Cells expressing SAS1B protein were detected with sheep anti-mouse IgG-FITC. (C–D) Intracellular and surface staining, respectively, of SAS1B in multiple human cancer cell lines and normal tissues. The identity of each cancer cell line is available in online supplemental table 2. FITC, fluorescein isothiocyanate; mAbs, monoclonal antibodies; MMMT, malignant mixed Mullerian tumor; PBMC, peripheral blood mononuclear cells; SAS1B, sperm acrosomal SLLP1 binding protein; SCC, squamous cell carcinoma.

We sought to identify a blocking peptide to confirm SAS1B specificity in subsequent assays. We mapped the epitope within SAS1B SV-A (431 amino acids). Western immunoreactivity to SAS1B, with bands ~50 kD, was observed, consistent with that seen in Coomassie staining and western blotting as described previously. The ~50 kD band was detected in cells transfected with residues 1–431, 24–431, or 32–431. It was not detected in cells transfected with residues 40–431, suggesting that SAS1B’s epitope is between amino acids 32–39 (figure 5A). Deletion constructs were developed to epitope map SV-C (436 amino acids), for which data suggest that the epitope is between amino acids 37–44 (data not shown). Epitope sequences for SV-A and SV-C were identical (GACGTSFP). Blocking (residues 24–42) and negative control (residues 377–394) peptides were synthesized.

10.1136/jitc-2023-008430.supp2 Supplementary data

### SAS1B expression in human cancer cell lines and normal tissues

Surface SAS1B expression was evaluated in multiple tumor cell lines of diverse histologies. Tumor cells were stained with SB2 and the control (amino acids 377–394) or blocking (amino acids 24–42) peptide, or with an isotype-matched IgG control. Strong SAS1B surface expression was detected in five different tumor cell lines with SB2 and the control peptide by flow cytometry. Specificity was confirmed by the blocking peptide, where signals were completely blocked (figure 5B, online supplemental table 2). Staining was performed in a range of cancer cell lines and normal tissues to gain a broader understanding of intracellular versus surface SAS1B expression. Intracellular expression of SAS1B cells was assessed by staining with SB2 after cells were fixed and permeabilized. This allowed SB2 to enter the cytoplasm and to bind intracellular SAS1B. In contrast, intact viable cells were used to assess SAS1B surface expression. On these viable cells, SB2 only stained surface SAS1B and was unable to enter the cytoplasm and bind intracellular SAS1B. Results represent averages and standard deviations of multiple assays where applicable, or without standard deviations in cases of single assays (figure 5C,D, online supplemental table 2). Intracellular SAS1B expression was demonstrated in evaluated cancer lines and some normal tissues. Despite finding intracellular SAS1B in multiple normal tissues, surface staining of these tissues was consistent with background signals and not blocked by the blocking peptide (figure 5D). This supports the hypothesis that SAS1B is not expressed on the surfaces of normal human cells. Surface staining of cancer cells demonstrated SAS1B on most lines at varying levels (figure 5D), consistent with the examples in figure 5B. There was variability in staining across multiple assays and cell lines. In each case, staining was completely blocked by the blocking peptide, confirming the specificity of SB2 (online supplemental table 2).

### SAS1B as a target for antibody-mediated cytotoxicity

**Figure 6:**
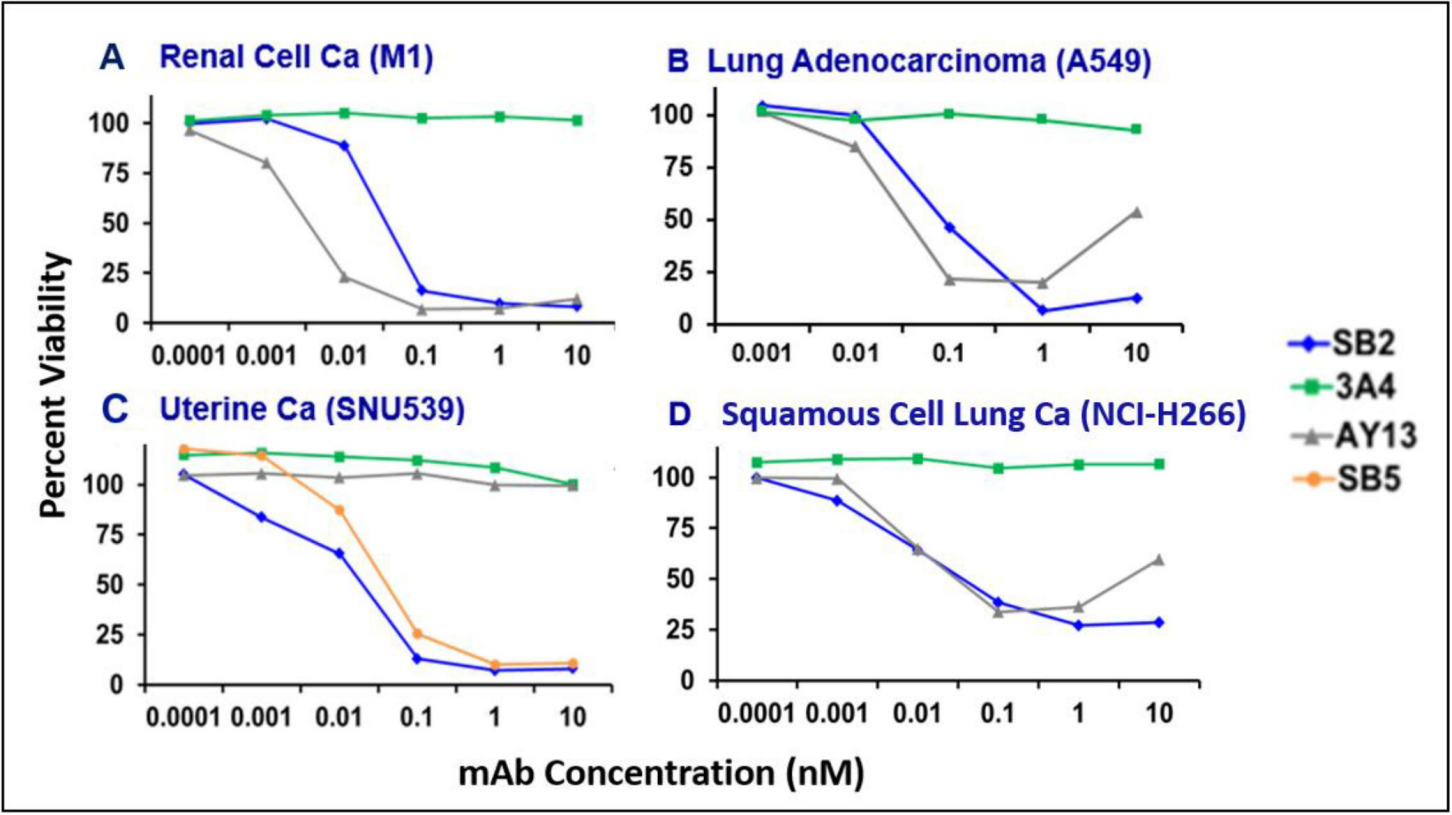
Four cancer cell lines were tested: M1 (renal cell carcinoma), A549 (lung adenocarcinoma), SNU539 (malignant mixed Mullerian tumor), and NCI-H226 (squamous cell carcinoma of the lung). These lines were tested with mAbs SB2 (blue lines), 3A4 (anti-Ca-binding tyrosine phosphorylation regulated protein) (negative control) (green lines), and AY13 (anti-epidermal growth factor receptor) (positive control) (gray lines). SNU539 was additionally tested with SB5 (orange line). ATP was quantitated as a measure of viability, from which percent lysis was calculated. mAbs, monoclonal antibodies.

SAS1B was tested as a potential target for ADC using Fab-αMFc-CL-DMDM. Cancer cell lines M1, A549, SNU539, and H226 were tested with mAbs SB2, 3A4 (anti-CABYR, negative control), and AY13 (anti-EGFR, positive control). SNU539 was additionally tested with SB5 mAb. ATP was quantitated as a measure of viability, from which percent lysis was calculated. Percent viability decreased with increasing SB2 ADC concentrations for all lines. Similar results were seen with SB5 ADC in SNU539. This group previously demonstrated similar findings in pancreatic cancer.^6^ In M1, A549, and H226, percent viability decreased with increasing AY13 ADC concentrations. Percent viability was not affected by increasing AY13 ADC concentrations in SNU539, which does not express surface EGFR. Increasing 3A4 ADC concentrations did not affect viability for any line. These results demonstrate strong dose-dependent cytotoxic killing with anti-SAS1B antibody comparable to AY13 (figure 6). Thus, SAS1B may serve as a potential target for antibody-mediated cytotoxicity in solid tumors.

## Discussion

Studies have shown oocyte SAS1B expression and its role during fertilization.^4^ SAS1B was not initially thought to be expressed in other tissues, but was found in uterine and pancreatic cancers.^5 6^ This paper evaluates SAS1B expression in a range of human cancers and normal human tissues outside of the ovary. IHC with a pAb demonstrated SAS1B expression in multiple cancers and none in other normal tissues, except for pancreatic islets. This raised concern for potential toxicity to pancreatic islets if SAS1B were targeted as a cancer therapeutic. We hypothesized that SAS1B expression differs in its location in cancer versus normal tissues, with surface expression being tumor-specific. To understand this selective expression and its potential use as a targeted therapy, mAbs were developed to confirm specificity. We developed and validated SB2 and SB5 mAbs as having the strongest reactivities and specificities to SAS1B, along with an effective blocking peptide from SAS1B. Using these mAbs, we demonstrated intracellular, not surface, SAS1B expression was detected in many normal cell types, at variable levels. However, surface expression was tumor-specific. SAS1B was tested in vitro as a potential immunotherapeutic target using an ADC. Strong dose-dependent cytotoxic killing was observed, indicating SAS1B’s potential use in antibody-mediated cytotoxicity.

To our knowledge, these are the first data demonstrating SAS1B expression in a variety of solid tumors, including cancers with high mortality rates. Our data demonstrate SAS1B surface expression by cancers of various histologies, but at varying levels, some of which may change over time. This suggests that environmental factors may influence expression. Previous work has shown SAS1B expression in human pancreatic cancer in situ, but without the ability to differentiate surface from intracellular expression.^6^ This data with three pancreatic cancer cell lines show surface expression on only one of the three lines tested (figure 5D). Future work should include evaluation of SAS1B surface expression in single cell suspensions of human tumor specimens to better understand the proportions of cancers, including pancreatic cancers, that express surface SAS1B. This future work will be aided by the SAS1B blocking peptide that we developed.

SAS1B expression in pancreatic islets, as demonstrated by IHC, raised concern for cross-reactivity and toxicity with employing SAS1B as a potential therapeutic cancer target. Subsequent flow cytometric analysis with mAbs confirmed expression by pancreatic islets; yet, expression was only intracellular, not surface, as hypothesized. Similarly, SAS1B was identified in high proportions of other normal tissues (kidney, cardiac myocytes, aortic endothelium) (figure 5C). Interestingly, for all normal tissues, SAS1B expression was only intracellular. None had cell surface expression. These data suggest that antibody-based therapies targeting SAS1B would likely be cancer-specific with low toxicity on normal tissues. Our prior work suggested SAS1B as a candidate immunotherapeutic target with ADCs in uterine and pancreatic cancers.^5 6^ This current work demonstrates that SAS1B is a candidate immunotherapeutic target with ADCs in additional cancers including renal cell carcinoma, lung adenocarcinoma, and squamous cell lung cancer (figure 6). Unlike SB2, which binds to intracellular SAS1B only after cell fixation and permeabilization, ADCs usually enter viable cells by receptor-mediated endocytosis. We did not directly study the mechanism of action of our anti-SAS1B ADC, but we expect the same to be the case with SB2 ADC. Once in the cell, the linker is cleaved by cathepsins,^12^ after which Duocarmycin diffuses to and enters the nucleus, where it inhibits DNA synthesis via adenosine alkylation, inducing apoptosis.^13^

The intent of this manuscript was to summarize the discovery work for SAS1B, which includes the identification of new SAS1B SV, the creation of SAS1B-specific antibodies and their characterization, and surface versus intracellular SAS1B expression and their specificity in normal and cancer cells. This manuscript is novel in that it demonstrates the specificity of SB2 and SB5 to SAS1B. Not only did this group develop those antibodies, but also it validated their specificity with the use of a blocking peptide. Strong SAS1B surface expression was detected in tumor cell lines, which was completely blocked with the blocking peptide. The most interesting finding within this paper is the tumor specificity of surface SAS1B expression. SAS1B expression was evaluated in multiple tumor cell lines and normal tissues of diverse histologies, which, to our knowledge, has not been reported before. SAS1B surface expression was demonstrated on a much wider range of solid tumors, including melanoma and breast, ovarian, renal, and lung cancers, which were not seen on normal cells. Our data support surface SAS1B as tumor-specific. T cells recognize major histocompatibility complex-associated peptide antigens that arise from intracellular proteins. Thus, SAS1B is likely not an ideal target for T-cell-based therapies or cancer vaccines that induce T cells. However, our findings support the exploration of antibody-based therapies, including ADCs and CAR-T cell therapy.

SAS1B is a first-in-class cancer-oocyte antigen. It is tempting to imagine similarities to cancer testis antigen. However, SAS1B’s expression in normal tissues highlights differences from cancer testis antigen and suggests that its expression in cancer is not due to reversion to a primitive state. This group has reported SAS1B’s role in sperm-oocyte interaction.^3 4^ This raises questions regarding why SAS1B is expressed in normal non-oocyte and cancer tissues and the roles it serves in those particular tissues. We suspect that the different SV may have selective surface versus intracellular localization that may explain the different localization in cancer versus normal cells. This selectivity may determine the specific functions they serve in normal versus cancer tissues. Future work will entail determining differences in SV, including each variant’s selectivity with regard to localization and function within normal versus cancer tissues. This will help determine the function of each variant and its relevance to target cancer therapy.

## Conclusions

SAS1B is expressed intracellularly in human cancers and some normal human tissues. However, SAS1B surface expression is unique to cancer. This selective expression may serve as a potential therapeutic cancer target in multiple cancers, many of which do not have effective therapies. The SB2 and SB5 mAbs to SAS1B offer promise for clinical application. Future work entails testing this ADC in vivo via systemic administration (intravenously and/or intraperitoneally), with hopes that it will prove to be effective and durable for a wide variety of malignancies. Additionally, future work includes a better understanding of the localization and function of SAS1B SV in normal and cancer tissues.

## Data Availability

Data are available upon reasonable request.
