# Supplementary material for: Cancer-oocyte SAS1B protein is expressed at the cell surface of multiple solid tumors and targeted with antibody-drug conjugates
Source: J Immunother Cancer. 2024 Mar 13;12(3):e008430. doi: 10.1136/jitc-2023-008430 (PMC10941168; doi:10.1136/jitc-2023-008430)
Supplement: Supplementary data [file jitc-2023-008430supp001.pdf]

Supplemental Table 1 – Human cell lines used in flow cytometry

Cell Line	Source	Product Number
A549 (lung adenocarcinoma)	American Type Culture Collection (ATCC), Gaithersburg, Maryland	CCL-185
MDA-MB-468 (breast adenocarcinoma)		HTB-132
BxPC3 (pancreatic adenocarcinoma)		CRL-1687
NCI-NCI-H226 (lung squamous cell carcinoma)		CRL-5826
Malme-3M (melanoma)		HTB-64
MPanc-96 (pancreatic adenocarcinoma)		CRL-2380
Panc366 (pancreatic adenocarcinoma)		IRB-HSR #13529
SKOV3 (ovarian adenocarcinoma)		HTB-77
SNU539 (malignant mixed Müllerian tumor)	Jae-Gahb Park, MD, PhD, Seoul National University, Seoul, South Korea	N/A
Aortic endothelium	PromoCell, Heidelberg, Germany	C-12271
Cardiac myocytes		C-12810
Fibroblasts	University of Virginia	IRB-HSR #10598
Pancreatic islets	Prodo Labs, Aliso Viejo, CA	HIR-001
Kidney (x2)	University of Virginia	IRB-HSR #10598
Skeletal muscle	PromoCell, Heidelberg, Germany	C-12530
Peripheral blood mononuclear cells (PBMC)	Virginia Blood Services, Richmond, VA	N/A
Spleen	University of Virginia	IRB-HSR #10598
Lymph node		
Lymphocyte		
