# Supplementary material for: Cancer-oocyte SAS1B protein is expressed at the cell surface of multiple solid tumors and targeted with antibody-drug conjugates
Source: J Immunother Cancer. 2024 Mar 13;12(3):e008430. doi: 10.1136/jitc-2023-008430 (PMC10941168; doi:10.1136/jitc-2023-008430)
Supplement: Supplementary data [file jitc-2023-008430supp002.pdf]

Supplemental Table 2 – Percent of live human normal and cancer cells expressing intracellular and surface SAS1B via flow cytometry

		INTRACELLULAR				SURFACE			
Cell Line	Cell Name	IgG (SD)	SB2 (SD)	SB2 + Control Peptide (a377-394) (SD)	SB2 + Blocking Peptide (aa24-42) (SD)	IgG (SD)	SB2 (SD)	SB2 + Control Peptide (a377-394) (SD)	SB2 + Blocking Peptide (aa24-42) (SD)
NCI-H226	SCC of the Lung	1.4	-	15.6	0.1	0.3	-	47.7	0.5
PANC366	Pancreatic AdenoCa 1	7.6 (10.5)	40.9	47.2 (4.8)	0.2 (0.3)	1.0	1.0	1.0	0.8
BxPC3	Pancreatic AdenoCa 2	0.7	47.0	46.4	0.8	0.5	0.8	0.8	0.4
mPANC96	Pancreatic AdenoCa 3	1.0	64.7	75.9	1.2	1.1	13.5	15.8	1.7
SNU539	MMMT	1.5 (0.6)	-	24.5 (10.5)	0.1 (0.0)	0.5 (0.6)	2.8	39.3 (51.5)	0.8 (0.2)
A549	Lung AdenoCa	1.0 (0.1)	-	37.6 (45.4)	0.4 (0.4)	1.1	-	2.0	1.7
Malme-3M	Melanoma	1.5	-	15.1	-	0.2	-	95.3	0.5
SKOV3	Ovarian AdenoCa	0.6 (0.5)	-	95.7 (3.5)	0.5 (0.4)	0.6 (0.3)	-	49.3 (62.4)	0.7 (0.1)
MD-MB-468	Breast AdenoCa	1.7 (1.8)	61.9 (23.4)	68.7 (23.0)	1.2 (0.8)	0.9 (0.3)	27.9 (24.4)	31.7 (30.0)	1.3 (0.6)
N/A	Aortic Endothelium	0.9 (0.1)	37.9	47.7 (10.1)	1.8 (1.2)	1.0 (0.6)	0.5 (0.1)	1.5 (1.2)	1.3 (0.9)
N/A	Cardiac Myocytes	0.8	-	44.8	1.8	1.0 (0.1)	0.8	1.1 (0.1)	0.9 (0.1)
N/A	Fibroblasts	0.8	8.1	4.9	1.5	1.6 (0.9)	1.7 (0.2)	1.7 (0.3)	1.0 (0.2)
N/A	Kidney 1	1.0 (0.1)	4.9 (1.7)	5.8 (1.7)	1.2 (0.2)	1.3 (1.7)	1.6 (1.3)	2.5 (1.0)	1.7 (0.7)
N/A	Kidney 2	0.9	-	82.6	1.0	0.9 (0.1)	1.0	2.5 (1.8)	0.8 (0.5)
N/A	Lymph Node	0.1	1.1	0.5	0.1	0.6	0.2	0.2	0.2
N/A	Lymphocytes	0.3	0.1	0.1	0.0	0.8	0.0	0.0	0.0
N/A	Pancreatic Islets	0.9 (0.1)	-	50.5 (25.7)	0.2 (0.1)	1.2 (0.4)	0.4 (0.4)	0.4 (0.4)	0.2 (0.0)
N/A	PBMC 1	0.5 (0.5)	2.6	1.4 (1.2)	0.1 (0.0)	0.7 (0.1)	0.1	0.1 (0.0)	0.1 (0.0)
N/A	PBMC 2	0.2 (0.1)	1.5 (0.7)	1.5 (0.9)	0.1 (0.0)	1.2 (0.5)	0.3 (0.2)	0.2 (0.2)	0.2 (0.3)
N/A	Skeletal Muscle	1.0 (0.4)	1.2	9.9 (12.3)	0.4 (0.5)	1.0 (0.1)	0.9 (0.1)	0.9 (0.0)	1.2 (0.1)

N/A	Spleen	0.1	0.3	0.3	0.1	1.4	0.2	0.1	0.2

- Not evaluable; SD – standard deviation; SCC – squamous cell carcinoma; AdenoCa – adenocarcinoma; MMMT – malignant mixed Mullerian tumor; PBMC – peripheral blood mononuclear cells
